# Effect of social integration on childbirth return among internal migrant pregnant women: a nationally representative study in China

**DOI:** 10.1186/s12913-020-05783-5

**Published:** 2020-10-07

**Authors:** Lulu Ding, Xinying Li, Xue Tang, Yuejing Feng, Yi Wang, Jiejie Cheng, Mei Sun, Chengchao Zhou

**Affiliations:** 1 Centre for Health Management and Policy, School of Public Health, Cheeloo College of Medicine, Shandong University, Jinan, 250012 China; 2 School of Public Health, Fudan University, Shanghai, 200032 China; 3 NHC Key Laboratory of Health Economics and Policy Research (Shandong University), 44 Wen-hua-xi Road, Jinan, 250012 Shandong China

**Keywords:** Internal migrant women, Childbirth return, Social integration

## Abstract

**Background:**

Social integration has been demonstrated to be associated with the health care use among migrants, but few studies have focused on migrant pregnant women. This study aims to explore the association between social integration and childbirth at woman’s hometown (childbirth return) of internal migrant pregnant women in China.

**Method:**

Using the data of “Monitoring Data of Chinese Migrants” in 2014, a total of 3412 internal migrant pregnant women were included in this study. Social integration was measured by economic integration, acculturation, and identification. The childbirth locations of internal migrant pregnant women were divided into current residency and the woman’s hometown. Univariate logistic regression and two multivariable logistic regression models were employed to assess the association between social integration and childbirth return among internal migrant pregnant women.

**Result:**

Our study finds that 24.56% of migrant pregnant women choose to have a childbirth return. As for social integration, those who have their own house (OR = 0.351 95% CI 0.207–0.595) in current residence, who have been staying in current residence for at least 5 years (OR = 0.449; 95% CI 0.322–0.626), and who are willing to stay in the current residence for a long time (OR = 0.731; 95% CI 0.537–0.995) are less likely to have a childbirth return. Apart from social integration, our results also show that those migrant pregnant women who are older, who have higher education level, who have at least two family members in current residence, with a migration reason of work and business, who have established health record in the current residency, and who were not covered by medical insurances, are less likely to have a childbirth return.

**Conclusion:**

Social integration is negatively associated with childbirth return among internal migrant pregnant women in China. To improve the utilization of maternal care services for migrant pregnant women in current residence, targeted policies should be made to improve social integration status for migrant pregnant women.

## Background

In China, the number of internal migrant population had increased from 230 million in 2011 to 244 million in 2017, which occupies a large proportion of nearly 18% of the total population [1]. The rapid expanding of migrant population scale promotes economic prosperity, but also brings a number of public health issues, including problems in infectious diseases, childhood immunization, mental health, maternal healthcare etc. [2–5] In the past decades, with the emergence of “family migration style”, more and more fertile women outmigrated with their husbands, and the number of migrant fertile women is on the rapid rise, accounting for over 30% of the total migrant population [6, 7]. Consequently, migrant maternal healthcare use is an important issue deserves attention.

From the perspective of inflow area, social integration is found to be an important factor associated with health service use among the migrants [8]. Social integration refers to the process during which migrants are incorporated into the social structure of the society in inflow areas. Although different scholars have different definitions and measurements of social integration, they all share certain dimensions: economic integration, acculturation, and identification [9–11]. A study by Kemppainen et al. about the health utilization by immigrants in Russia found that stronger social integration predicted less frequent return for health care utilization [12]. In some countries, such as South Korea, special policies have been developed to improve social integration, so as to promote health status among migrant fertile women [13].

Pregnancy is a critical period, not only for the pregnant woman and her unborn child, but also for her whole family. Mobility during pregnancy is found to be linked to poor health outcomes of pregnant mothers and infants or unborn child [14]. Residential instability during pregnancy brings environmental change which was found to be associated with congenital anomalies [15–17]. There are some other studies indicating that mobility during pregnancy may lead to decreased use of maternal healthcare services due to less knowledge and information, or other barriers to accessibility of health service [18, 19]. Some previous studies found that mobility during pregnancy was detrimental to healthcare provision, which was due to the failure to address the scale and traits of the migrant pregnant women and their specific demands [20, 21]. A series of policies, such as promoting the equalization of public health services^,^ [22–24] were developed to enable the migrants in the inflow areas to receive the same medical and public health services as local permanent residents. In addition, the China Women’s Development Program 2011–2020 clearly states that migrant women should receive the same health care services as the women in the inflow areas [25]. Even so, previous studies have demonstrated that the migrants have poorer access to health care services in the inflow areas than that in local permanent residents [26]. As a coping style, some migrants would like to return their hometown for health care use. A previous study by Bergmark et al. showed that 46% Mexican immigrants reported they had a close friend or relative returned to Mexico from U.S. for health care use [27]. A similar study by Jiang also found that about one-fourth of the respondents have visited their home country for medical care since their migration to the US [28]. Similarly, there are also some relevant studies in China. A study by Song reported that 37.23% of 118 hospitalized migrants in Guangzhou had returned to their hometown for medical care [29]. Another study in Beijing reported that 63.16% of 114 migrant returned to their hometown for in-patient services [30]. Of the reasons for their returning hometown for health care use, preferring medical style of homeland or having social ties with homeland are the common ones that facilitate their return [31, 32].

In China, there are some studies focusing on migrants and social integration status, but mainly concentrating on the association between social integration and physical or psychological well-being of migrants [33, 34]. Few studies explored the migrants’ return for healthcare use, and also very few studies focused on the association between social integration and healthcare use among the migrants. To date, no studies have explored the association between social integration and returning their hometown for childbirth (childbirth return) among internal migrant women in China. To remedy this situation, this study aims to explore the association between social integration and childbirth return among internal migrant pregnant women. To do so, we have several specific objectives. First, we will estimate the prevalence of childbirth return among migrant pregnant women. Second, we will explore the association between the social integration and childbirth return among internal migrant pregnant women in China.

## Methods

### Data source

The data were obtained from the “2014 National Internal Migrant Population Dynamic Monitoring Survey (NIMPDMS)”, which was conducted by the National Health and Family Planning Commission of China. Inclusion criteria for the survey were adults living in the inflow areas for 1 month or longer without local Hukou registration (Hukou is the household registration system in China, which was divided into rural and urban in this study) and aged between 15 and 59 years as of May 2015. It was a nationally representative survey which was conducted in 32 provinces, municipalities, autonomous regions, and migrant gathering areas in Xinjiang Production and Construction Corps. A total of 431 cities were selected as sample sites. The survey used a stratified multi-stage sampling method with a probability-proportional-to-size (PPS) approach to select participants [35]. Face-to-Face interviews were conducted by trained health staff in sampling communities. It took about 1 h for the interviewers to conduct each interview. The survey included respondents’ demographic characteristics, their family members, employment status, housing, health care, marital status, obstetrical status. The total sample size was 206,000 people in 2014, and the criteria of the sample selection in this study was that who gave birth in the past year before the survey. Finally, 3412 eligible internal migrant pregnant women were included in the analysis, and the sampling process is presented in the Additional file 1: Figure S1.

### Variables

#### Dependent variable

The participants in this study are filtered by two questions: “Have you had used in-patient service in the past 12 months?” If the answer is “yes”, then a second question of “What is the leading reason for your in-patient service?” Those women whose answer was “Hospital childbirth” were included in this study. The dependent variable of this study was defined by asking the respondents “Where was your hospital childbirth?” The answer was divided into two types, “returning their hometown”, and “in the current residence”.

#### Independent variables

##### Social integration

Based on the previous literature, combining with the questionnaire used in the survey, the social integration in this study was mainly measured from the dimensions of economic integration, acculturation, and identification. Factors including employment status (unemployment and employment), household monthly income, and housing in the current residence (rent or borrow, own, others) were used to measure the dimension of economic integration. Duration of stay in current residence (5 years and below, and more than 5 years) was used to measure the dimension of acculturation. Willingness to stay for a long time (no and yes) was used to measure the dimension of identification. Here, we used the quartile method to divide household monthly income into four levels, Q1, Q2, Q3, and Q4. Quartile1(Q1) is the lowest (poorest) group and Quartile4(Q4) is the highest (richest) group.

##### Social demographic characteristics

Social demographic characteristics include age, ethnic group, education level, family members in current residence, Hukou, migration range, the reason for migration, health record in current residence, and medical insurance. The participants’ age was described by mean and standard deviation. Other demographic characteristics were categorized as follows: ethnic group including minority ethnic (including Mongolian, Manchu, Hui, Tibetan and other minority ethnic groups) and Hans, education level (middle school and below, high school and above), family members in current residence (one or two, three, four, more than four), migration range (inter-provincial and intra-provincial), the reason for migration (work or business, accompany, and others),whether to establish health record in current residence (no and yes), and medical insurance (no and yes).

### Statistical analysis

All statistical analysis are performed using SPSS 24.0 and Stata 14.2. Percentages are used to describe the variables, and univariate logistic regression is used to explore the association between all variables and childbirth return. Two multivariable logistic regression models with an enter method are employed to examine the associations between social integration and childbirth return among migrant women. In model 1, we only include the indicators of the social integration. In model 2, we include some other potential confounders so as to examine whether social integration had significant influence on the choice of childbirth site when controlling for other factors. Those variables that have been proved to be associated with the health service use among the migrants (e.g., family members in current residence, Hukou, establishment of health records) and also collected in this study were included as control variables [36, 37]. As for the explanatory variables, because social integration is a dynamic, multi-dimensional concept, we used the indicators which have been applied in many previous studies that are mostly cited and recognized to measure social integration in this study [10, 11, 33, 38]. According to the work book of the NIMPDMS in 2014, the weights of this study are only derived from strata and non-response weight, and all provincial urban belt and key cities were stratified, [35] and strata were finally determined by using the stratified multistage random sampling method. Within these, townships were selected as first-stage sampling units, and villages were selected from those townships. Finally, the individual respondents were selected from the selected village committee. We cannot cluster by townships because the information of townships is not available in the 2014 NIMPDMS. Thus, all analyses are conducted by incorporating strata and clustering in villages, and sampling weights are included.

## Results

### The demographic characteristics

Table 1 shows the basic information of the 3412 migrants. About 24.56% of internal migrant pregnant women chose to have a childbirth return. The average age of the migrant pregnant women is 29.04 years, with a standard deviation of 4.35 years. The majority of the women are from rural areas (78.74%),Hans (93.50%), have an education level of middle school and below (48.47%), inter-provincial migrants (32.83%), with a migration reason for work and business (70.37%), covered by medical insurance (83.46%), and did not establish health record in the current residence (73.72%). According to the result of univariate logistic regression, factors including age, ethnic group, education level, family members in current residence, Hukou, migration reason, establishment of health record, and medical insurance are significantly associated with childbirth return among migrant pregnant women.

**Table 1:** The demographic characteristics of the sampling internal migrant pregnant women in China, 2014

Variables	N(%)	Childbirth return		Unadjusted model		
		No	Yes	OR	95%CI	*P*
Observations	3412 (100)	2583 (75.44)	829 (24.56)			
Age	29.04 ± 4.35^a^	2583 (75.44)	829 (24.56)	0.941	0.911–0.972	0.000
Ethnic group						
Minority ethnic^b^	294 (6.50)	243 (82.91)	51 (17.09)	1.0		
Hans	3118 (93.50)	2340 (74.92)	778 (25.08)	1.624	0.980–2.690	0.060
Education level						
Middle school and below	1728 (48.47)	1239 (69.30)	489 (30.70)	1.0		
High school	759 (24.45)	575 (75.93)	184 (24.07)	0.716	0.520–0.984	0.040
Above high school	925 (27.08)	769 (85.97)	156 (14.03)	0.368	0.267–0.508	0.000
Family members in current residence						
≤ 2	290 (9.51)	130 (46.28)	160 (53.72)	1.0		
3	1951 (51.81)	1494 (74.79)	457 (25.21)	0.290	0.190–0.444	0.000
4	930 (30.24)	749 (82.77)	181 (17.23)	0.179	0.113–0.284	0.000
> 4	241 (8.44)	210 (85.94)	31 (14.06)	0.141	0.066–0.302	0.000
Hukou						
Rural	2707 (78.74)	1994 (72.91)	713 (27.09)	1.0		
Urban	705 (21.26)	589 (84.79)	116 (15.21)	0.483	0.351–0.666	0.000
Migration range						
Inter-provincial^c^	1730 (32.83)	1301 (73.91)	429 (26.09)	1.0		
Intra-provincial^d^	1682 (67.17)	1282 (76.18)	400 (23.82)	0.886	0.667–1.175	0.401
Migration reason						
Work and business	2074 (70.37)	1612 (78.78)	462 (21.22)	1.0		
Accompany	1144 (26.49)	803 (66.33)	341 (33.67)	1.885	1.460–2.432	0.000
Others	194 (3.14)	168 (77.41)	26 (22.59)	1.083	0.437–2.686	0.863
Establishment of health records						
No	2476 (73.72)	1809 (72.53)	667 (27.47)	1.0		
Yes	936 (26.28)	774 (83.57)	162 (16.43)	0.519	0.359–0.751	0.001
Social medical insurance status						
Uninsured	470 (16.54)	386 (84.23)	84 (15.77)	1.0		
Insured	2942 (83.46)	2197 (73.69)	745 (26.31)	1.907	1.245–2.920	0.003

^a^Presented as mean ± standard deviation

^b^Minority ethnic including Mongolian, Manchu, Hui, Tibetan and other minority ethnic groups

^c^Inter-provincial refers to Chinese internal migrant who migrated just within one province,including among different cities and counties within a province

^d^Intra-provincial refers to Chinese internal migrant who migrated from one province to another province

### Social integration

Social integration is measured by indicators of employment status, household monthly income, housing, duration of stay in current residence, and willingness to stay for a long time (Table 2). Of the respondents, 1408(44.80%) participants are employed. Regarding to household monthly income, the proportions of childbirth return of four income groups from low to high are 22.39, 18.49, 32.29, 26.83%, respectively. In addition, 76.86% of participants’ house are rented or borrowed, only 18.90% of participants have their own house in the current residence. Moreover, the rate of childbirth return among those whose housing is their own is 8.09%, compared to 27.70% of those whose housing is rented or borrowed. Most of migrant pregnant women in the study have stayed in current residence for less than 5 years (65.70%). About 61.95% of participants clearly expressed their willingness to stay for a longer time in current residence. Of whom, the rate of childbirth return is 18.38%, compared to a rate of 34.63% among those who do not want to stay in current residence for a long time. According to the result of univariate logistic regression, factors including household monthly income, housing type, duration of stay in current residence, and willingness to stay in the current residence for long time are significantly associated with childbirth return among migrant pregnant women.

**Table 2:** Childbirth return across different type of social integration status among internal migrant pregnant women in China, 2014

Variables		Childbirth return		Unadjusted model		
	N(%)	No	Yes	OR	95%CI	*P*
Observations	3412 (100)	2583 (75.44)	829 (24.56)			
*Economic integration status*						
Employment status						
No	2004 (55.20)	1526 (73.70)	478 (26.30)	1.0		
Yes	1408 (44.80)	1057 (77.57)	351 (22.43)	0.810	0.638–1.030	0.085
Household monthly income ^a^						
Q1	1028 (22.39)	748 (72.23)	280 (27.77)	1.0		
Q2	682 (18.49)	506 (72.55)	176 (27.45)	0.984	0.688–1.407	0.930
Q3	1016 (32.29)	770 (74.68)	246 (25.32)	0.881	0.645–1.204	0.427
Q4	686 (26.83)	559 (81.01)	127 (18.99)	0.610	0.411–0.905	0.014
Housing type						
Rent or borrow	2426 (76.86)	1753 (72.30)	673 (27.70)	1.0		
Own	847 (18.90)	752 (91.91)	95 (8.09)	0.230	0.148–0.356	0.000
Others	1394.23)	78 (58.72)	61 (41.28)	1.835	1.086–3.100	0.023
*Acculturation*						
Duration of stay in current residence						
< 5 years	2337 (65.70)	1663 (69.64)	674 (30.36)	1.0		
≥ 5 years	1075 (34.30)	920 (86.54)	155 (13.46)	0.357	0.265–0.480	0.000
*Identification*						
Willingness to stay for a long time						
No	1197 (38.05)	776 (65.37)	421 (34.63)	1.0		
Yes	2215 (61.95)	1807 (81.62)	408 (18.38)	0.425	0.323–0.560	0.000

^a^Quartile 1 (Q1) is the lowest (poorest) and Quartile 4 (Q4) is the highest (richest) group

### The association between social integration status and childbirth return

We present our results in two models so that we can better explore the association between social integration and childbirth return (Table 3). In Model 1, we only include social integration status. The results show that the association between social integration and the childbirth return among migrant pregnant women is statistically significant. In detail, the migrant pregnant women who do not have their own house in the current residence (*P* = 0.000,*OR =* 0.351), have shorter duration of stay in current residence (*P* = 0.000,*OR =* 0.449), have no willingness to stay in the current residence for a long time (*P* = 0.046, *OR =* 0.731) are more likely to have a childbirth return. Even after all other variables were controlled, as shown in Model 2, the association between social integration and the childbirth return of migrant pregnant women is still significant.

**Table 3:** Association between social integration and childbirth return among internal migrant women in China, 2014

Variables	Regression Model 1			Regression Model 2		
	OR	95%CI	*P*	OR	95%CI	*P*
Household monthly income ^a^						
Q1	1.0			1.0		
Q2	1.050	0.718–1.535	0.801	1.040	0.700–1.547	0.845
Q3	1.021	0.731–1.426	0.905	1.190	0.826–1.713	0.350
Q4	0.971	0.626–1.505	0.894	1.402	0.886–2.218	0.149
Housing type						
Rent or borrow	1.0			1.0		
Own	0.311	0.196–0.493	0.000	0.351	0.207–0.595	0.000
Others	2.038	1.180–3.521	0.011	1.608	1.007–2.568	0.047
Duration of stay in current residence						
< 5 years	1.0			1.0		
≥ 5 years	0.413	0.301–0.566	0.003	0.449	0.322–0.626	0.000
Willingness to stay for a long time						
No	1.0			1.0		
Yes	0.634	0.471–0.853	0.003	0.731	0.537–0.995	0.046
Age				1.007	0.974–1.040	0.694
Education level						
Middle school and below				1.0		
High school				0.735	0.531–1.019	0.065
Above high school				0.369	0.238–0.572	0.000
Family members in current residence						
≤ 2				1.0		
3				0.271	0.170–0.432	0.000
4				0.146	0.088–0.240	0.000
> 4				0.122	0.055–0.269	0.000
Hukou						
Rural				1.0		
Urban				0.871	0.567–1.337	0.527
Migration reason						
Work and business				1.0		
Accompany				1.748	1.311–2.332	0.000
Others				1.128	0.438–2.905	0.803
Establishment of health records						
No				1.0		
Yes				0.678	0.470–0.978	0.038
Social medical insurance status						
Uninsured				1.0		
Insured				2.048	1.280–3.278	0.003

^a^Quartile 1 (Q1) is the lowest (poorest) and Quartile 4 (Q4) is the highest (richest) group

Besides, factors including age, education level, family members in current residence, the reason for migration, whether established health record in the current residence, and social medical insurance are also associated with childbirth return (Table 3).

## Discussion

The current study finds that24.56% of the internal migrant pregnant women have childbirth return, which is lower than that among the migrant pregnant women in Beijing (59.2%) [39]. There are several possible reasons why the childbirth return rate among migrant women in Beijing is higher than that of the national average level. First, Beijing is the capital of China, and high-quality maternal healthcare service resources are intensively allocated in Beijing, which attracts a large number of pregnant women from all over the country to use maternal care, including childbirth, in Beijing [40]. This thus the accessibility to maternal care is reduced for the migrant women. Second, medical expenses in Beijing are higher than the average level in the whole country [41]. It is hard for the migrants with low income to use high-expense childbirth services in Beijing.

This study indicates that over 70% of the migrant pregnant women give birth in the current residence, and this percentage is still on the rise. This is a positive trend but would probably increase the burden of post-partum care for the migrant mothers and babies, especially for the healthcare practitioners in the community health centers in the inflow areas. This finding implies for the healthcare practitioners in the inflow areas to establish health records for migrant pregnant women, so as to provide continuous and timely post-partum care for the migrant mothers and babies. In addition, the primary healthcare practitioners should also carry out comprehensive health education related to the potential risk of residential instability during pregnancy and importance of the post-partum care for the mothers and babies, to further increase the percentage of childbirth in the current residence and also a better postpartum recovery among the migrant pregnant women. As a long-term countermeasure, to establish a more integrated pregnancy record system for migrant pregnant women, and then the migrant pregnant women starting care in inflow site could continue accessing care if they return to their hometown, so as to improve health outcomes whether they give childbirth in the inflow area or at their hometown.

Some previous studies found that social integration was beneficial for some health.

outcomes among migrants [33, 42, 43]. This study also demonstrates an association between social integration and childbirth return among internal migrant women in China. The social integration is found to be negatively associated with childbirth return. The higher the level of the social integration is, the less possibility the migrant fertile women have a childbirth return. This association is multifaceted, lies in the dimensions of economic integration, acculturation, and identification. This finding implies that improving the localization situation (local adaption) and also income so as to promote the social integration might be helpful for the migrant women to use maternal care in the inflow areas.

As for the dimension in the economic integration, this study finds that migrant women who do not have their own house in the inflow areas are more likely to have childbirth return. For migrant pregnant women who do not have their own house in the inflow areas, and living in renting houses with poor sanitary and environmental conditions, they are more willing to return their hometown to get better home care when giving a birth to a child. In addition, those who have a house in the inflow areas will be easier to get formal or informal maternal postpartum care, which is similar to some previous studies [44].

Acculturation and identification are both significantly associated with the childbirth return among migrant women. The results show that the shorter the migrant women stay in inflow areas, the more likely they are to have a childbirth return. According to a study by Zhou, [10] the length of the duration of stay in the inflow areas, to a certain extent, can reflect the social adaptation of the migrants. That is to say, the migrant women who stay for a longer time, may have higher degree in social adaption, and also be more likely to get social support in the inflow areas, which are useful for the migrant women to use maternal care in the inflow areas [45]. With respect to self-identity, this study finds that migrant pregnant women who are willing to live in inflow areas for a long time are less likely to have a childbirth return. Identification can comprehensively reflect the integration status of migrants to the local population [36]. The migrant women with lower identification may have poorer adaptability to the local residents. As a result, the migrant pregnant women are more likely to return their hometown for childbirth. Measures to improve the identification are helpful for the migrant women to use maternal care in the current residence. For example, early antenatal classes for migrant women would assist them acquire supports in the current residence, which would be useful to make the migrant pregnant women feel more comfortable to give birth in the current residence.

Apart from social integration status, some other factors are also found to be associated with childbirth return among migrant women. The migrant pregnant women who have a lower education level are more likely to have childbirth returns. This might be due to that higher education can enable migrant women to acquire more maternal health care knowledge, including the risk of mobility during pregnancy [46]. As shown in the study, the more family members live together in the inflow areas, the less likely the migrant women are to return their hometown for childbirth. The reason might be that the large family size in current residence, to some extent, may indicate that the migrants have gradually adapted to the local life and culture. In addition, our study finds that the migrant pregnant women who outmigrate for accompany (the women who migrate because their husband or partner), who do not establish health records in the current residence, and who are covered by any kind of social medical insurance scheme are more likely to have childbirth returns.

This study has some limitations. First, the data of the study were based on self-reported measures which might lead to recall bias. Secondly, due to the limitations of the questionnaire, we only selected some indicators of economic integration, acculturation, and identification, and some other potential factors (e.g., language) were not included, which may not comprehensively reflect social integration status of the internal migrant pregnant women. Finally, we did not use qualitative methods to explore the push and pull factors for women’s choice of childbirth place, as well as an in-depth study of the postnatal outcomes of migrant pregnant women who give birth in hometown or in inflow areas, which would be remedied in the follow-up studies.

## Conclusion

This study finds that nearly a quarter of the migrant women return their hometown for childbirth. A significant association between social integration status and childbirth return is demonstrated among internal migrant women in China, especially lies in the dimensions of economic integration, acculturation, and identification. Migrant women with low social integration (with low income, the duration of stay in the inflow area less than 5 years, unwilling to stay for a long time) are more likely to return their hometown to give birth. This finding implies for interventions to improve the localization situation might be helpful for the migrant women to use maternal care in the inflow areas. Based on these findings, targeting promotion in social integration among migrant pregnant women, giving full consideration to their difference in culture, identification and other aspects, should be given to improve the utilization rate of maternal care in the current residence. Early antenatal classes for migrant women would assist them acquire supports in the current residence, which would be useful to make the migrant pregnant women feel more comfortable to give birth in the current residence. This study also finds some other factors, including education, family members in current residence, the reason for migration, health record, and social medical insurance, are associated with the choice of childbirth location.

## Supplementary information

**Additional file 1: Figure S1.** Flowchart of selecting process for the participants.

## Data Availability

The data used in this study are from the National Health and Family Planning Commission of China. The authors cannot share the data without approval from the National Health and Family Planning Commission of China. Those who want to request the data are encouraged to contact the corresponding author of this paper.
