# Supplementary material for: Effect of social integration on childbirth return among internal migrant pregnant women: a nationally representative study in China
Source: BMC Health Serv Res. 2020 Oct 7;20:918. doi: 10.1186/s12913-020-05783-5 (PMC7541329; doi:10.1186/s12913-020-05783-5)
Supplement: Supplementary file 1 — Additional file 1: Figure S1. Flowchart of selecting process for the participants. [file 12913_2020_5783_MOESM1_ESM.docx]

In the past 12months, have you had used in-patient service?

N=6503

Is childbirth the reason for your hospitalization?

Where was your hospital childbirth?

N=3800

N=829

N=2583

Yes

Yes

Hometown

Current residence

N=388

No answer or in other places

Figure 1. Flowchart of selecting process for the participants
